# Supplementary material for: A fault-intrusion-tolerant system and deadline-aware algorithm for scheduling scientific workflow in the cloud
Source: PeerJ Comput Sci. 2021 Nov 2;7:e747. doi: 10.7717/peerj-cs.747 (PMC8576554; doi:10.7717/peerj-cs.747)
Supplement: Supplemental Information 6 [file peerj-cs-07-747-s006.zip › Myproject1.1/docs/org/workflowsim/WorkflowEngine.html]

WorkflowEngine


JavaScript is disabled on your browser.


- Overview
- Package
- Class
- Use
- Tree
- Deprecated
- Index
- Help

- Prev Class
- Next Class

- Frames
- No Frames

- All Classes

- Summary:
- Nested |
- Field |
- Constr |
- Method

- Detail:
- Field |
- Constr |
- Method


org.workflowsim

## Class WorkflowEngine

- java.lang.Object
- - org.cloudbus.cloudsim.core.SimEntity
  - - org.workflowsim.WorkflowEngine

- All Implemented Interfaces:
  :   java.lang.Cloneable

  ---

    

  ```
  public class WorkflowEngine
  extends SimEntity
  ```

  WorkflowEngine represents a engine acting on behalf of a user. It hides VM
  management, as vm creation, submission of cloudlets to this VMs and
  destruction of VMs.

  Since:
  :   WorkflowSim Toolkit 1.0

- - ### Field Summary

    Fields

    | Modifier and Type | Field and Description |
    | --- | --- |
    | `protected java.util.List<? extends Cloudlet>` | `jobsList` The job list. |
    | `protected java.util.List<? extends Cloudlet>` | `jobsReceivedList` The job received list. |
    | `protected int` | `jobsSubmitted` The job submitted. |
    | `protected java.util.List<? extends Cloudlet>` | `jobsSubmittedList` The job submitted list. |
    | `protected java.util.List<? extends Vm>` | `vmList` |

    - ### Fields inherited from class org.cloudbus.cloudsim.core.SimEntity

      `FINISHED, HOLDING, RUNNABLE, WAITING`
  - ### Constructor Summary

    Constructors

    Constructor and Description

    `WorkflowEngine(java.lang.String name)`

    Created a new WorkflowEngine object.

    `WorkflowEngine(java.lang.String name,
    int schedulers)`
  - ### Method Summary

    Methods

    | Modifier and Type | Method and Description |
    | --- | --- |
    | `void` | `bindSchedulerDatacenter(int datacenterId)` Binds a datacenter to the default scheduler (id=0) |
    | `void` | `bindSchedulerDatacenter(int datacenterId, int schedulerId)` Binds a scheduler with a datacenter. |
    | `java.util.List<? extends Vm>` | `getAllVmList()` |
    | `<T extends Cloudlet>  java.util.List<T>` | `getJobsList()` Gets the job list. |
    | `<T extends Cloudlet>  java.util.List<T>` | `getJobsReceivedList()` Gets the job received list. |
    | `<T extends Cloudlet>  java.util.List<T>` | `getJobsSubmittedList()` Gets the job submitted list. |
    | `WorkflowScheduler` | `getScheduler(int schedulerId)` Gets the scheduler . |
    | `int` | `getSchedulerId(int index)` Gets the scheduler id list. |
    | `java.util.List<java.lang.Integer>` | `getSchedulerIds()` Gets the scheduler id. |
    | `java.util.List<WorkflowScheduler>` | `getSchedulers()` Gets the schedulers. |
    | `<T extends Vm>  java.util.List<T>` | `getVmList()` Gets the vm list. |
    | `void` | `processEvent(SimEvent ev)` Processes events available for this Broker. |
    | `protected void` | `processJobReturn(SimEvent ev)` Process a job return event. |
    | `protected void` | `processJobSubmit(SimEvent ev)` Process a submit event |
    | `protected void` | `processOtherEvent(SimEvent ev)` Overrides this method when making a new and different type of Broker. |
    | `protected void` | `processResourceCharacteristicsRequest(SimEvent ev)` Process a request for the characteristics of a PowerDatacenter. |
    | `void` | `shutdownEntity()` This method is invoked by the `Simulation` before the simulation finishes. |
    | `void` | `startEntity()` This method is invoked by the `Simulation` class when the simulation is started. |
    | `void` | `submitCloudletList(java.util.List<? extends Cloudlet> list)` This method is used to send to the broker the list of cloudlets. |
    | `protected void` | `submitJobs()` Submit jobs to the created VMs. |
    | `void` | `submitVmList(java.util.List<? extends Vm> list)` |
    | `void` | `submitVmList(java.util.List<? extends Vm> list, int schedulerId)` This method is used to send to the broker the list with virtual machines that must be created. |

    - ### Methods inherited from class org.cloudbus.cloudsim.core.SimEntity

      `cancelEvent, clone, getEventBuffer, getId, getName, getNextEvent, getNextEvent, getState, numEventsWaiting, numEventsWaiting, pause, run, schedule, schedule, schedule, schedule, scheduleFirst, scheduleFirst, scheduleFirst, scheduleFirst, scheduleFirstNow, scheduleFirstNow, scheduleFirstNow, scheduleFirstNow, scheduleNow, scheduleNow, scheduleNow, scheduleNow, selectEvent, send, send, send, send, sendNow, sendNow, sendNow, sendNow, setEventBuffer, setId, setState, waitForEvent`
    - ### Methods inherited from class java.lang.Object

      `equals, finalize, getClass, hashCode, notify, notifyAll, toString, wait, wait, wait`

- - ### Field Detail


    - #### jobsList

      ```
      protected java.util.List<? extends Cloudlet> jobsList
      ```

      The job list.


    - #### jobsSubmittedList

      ```
      protected java.util.List<? extends Cloudlet> jobsSubmittedList
      ```

      The job submitted list.


    - #### jobsReceivedList

      ```
      protected java.util.List<? extends Cloudlet> jobsReceivedList
      ```

      The job received list.


    - #### jobsSubmitted

      ```
      protected int jobsSubmitted
      ```

      The job submitted.


    - #### vmList

      ```
      protected java.util.List<? extends Vm> vmList
      ```
  - ### Constructor Detail


    - #### WorkflowEngine

      ```
      public WorkflowEngine(java.lang.String name)
                     throws java.lang.Exception
      ```

      Created a new WorkflowEngine object.

      Parameters:
      :   `name` - name to be associated with this entity (as required by
          Sim\_entity class from simjava package)

      Throws:
      :   `java.lang.Exception` - the exception


    - #### WorkflowEngine

      ```
      public WorkflowEngine(java.lang.String name,
                    int schedulers)
                     throws java.lang.Exception
      ```

      Throws:
      :   `java.lang.Exception`
  - ### Method Detail


    - #### submitVmList

      ```
      public void submitVmList(java.util.List<? extends Vm> list,
                      int schedulerId)
      ```

      This method is used to send to the broker the list with virtual machines
      that must be created.

      Parameters:
      :   `list` - the list


    - #### submitVmList

      ```
      public void submitVmList(java.util.List<? extends Vm> list)
      ```


    - #### getAllVmList

      ```
      public java.util.List<? extends Vm> getAllVmList()
      ```


    - #### submitCloudletList

      ```
      public void submitCloudletList(java.util.List<? extends Cloudlet> list)
      ```

      This method is used to send to the broker the list of cloudlets.

      Parameters:
      :   `list` - the list


    - #### processEvent

      ```
      public void processEvent(SimEvent ev)
      ```

      Processes events available for this Broker.

      **Specified by:**
      :   `processEvent` in class `SimEntity`

      Parameters:
      :   `ev` - a SimEvent object


    - #### processResourceCharacteristicsRequest

      ```
      protected void processResourceCharacteristicsRequest(SimEvent ev)
      ```

      Process a request for the characteristics of a PowerDatacenter.

      Parameters:
      :   `ev` - a SimEvent object


    - #### bindSchedulerDatacenter

      ```
      public void bindSchedulerDatacenter(int datacenterId,
                                 int schedulerId)
      ```

      Binds a scheduler with a datacenter.

      Parameters:
      :   `datacenterId` - the data center id
      :   `id` - the scheduler id


    - #### bindSchedulerDatacenter

      ```
      public void bindSchedulerDatacenter(int datacenterId)
      ```

      Binds a datacenter to the default scheduler (id=0)

      Parameters:
      :   `datacenterId` - dataceter Id


    - #### processJobSubmit

      ```
      protected void processJobSubmit(SimEvent ev)
      ```

      Process a submit event

      Parameters:
      :   `ev` - a SimEvent object


    - #### processJobReturn

      ```
      protected void processJobReturn(SimEvent ev)
      ```

      Process a job return event.

      Parameters:
      :   `ev` - a SimEvent object


    - #### processOtherEvent

      ```
      protected void processOtherEvent(SimEvent ev)
      ```

      Overrides this method when making a new and different type of Broker.
      This method is called by `#body()` for incoming unknown tags.

      Parameters:
      :   `ev` - a SimEvent object


    - #### submitJobs

      ```
      protected void submitJobs()
      ```

      Submit jobs to the created VMs.


    - #### shutdownEntity

      ```
      public void shutdownEntity()
      ```

      **Description copied from class: `SimEntity`**

      This method is invoked by the `Simulation` before the simulation finishes. If you want
      to save data in log files this is the method in which the corresponding code would be placed.

      **Specified by:**
      :   `shutdownEntity` in class `SimEntity`


    - #### startEntity

      ```
      public void startEntity()
      ```

      **Description copied from class: `SimEntity`**

      This method is invoked by the `Simulation` class when the simulation is started. This
      method should be responsible for starting the entity up.

      **Specified by:**
      :   `startEntity` in class `SimEntity`


    - #### getJobsList

      ```
      public <T extends Cloudlet> java.util.List<T> getJobsList()
      ```

      Gets the job list.

      Type Parameters:
      :   `T` - the generic type

      Returns:
      :   the job list


    - #### getJobsSubmittedList

      ```
      public <T extends Cloudlet> java.util.List<T> getJobsSubmittedList()
      ```

      Gets the job submitted list.

      Type Parameters:
      :   `T` - the generic type

      Returns:
      :   the job submitted list


    - #### getJobsReceivedList

      ```
      public <T extends Cloudlet> java.util.List<T> getJobsReceivedList()
      ```

      Gets the job received list.

      Type Parameters:
      :   `T` - the generic type

      Returns:
      :   the job received list


    - #### getVmList

      ```
      public <T extends Vm> java.util.List<T> getVmList()
      ```

      Gets the vm list.

      Type Parameters:
      :   `T` - the generic type

      Returns:
      :   the vm list


    - #### getSchedulers

      ```
      public java.util.List<WorkflowScheduler> getSchedulers()
      ```

      Gets the schedulers.

      Type Parameters:
      :   `T` - the generic type

      Returns:
      :   the schedulers


    - #### getSchedulerIds

      ```
      public java.util.List<java.lang.Integer> getSchedulerIds()
      ```

      Gets the scheduler id.

      Type Parameters:
      :   `T` - the generic type

      Returns:
      :   the scheduler id


    - #### getSchedulerId

      ```
      public int getSchedulerId(int index)
      ```

      Gets the scheduler id list.

      Type Parameters:
      :   `T` - the generic type

      Returns:
      :   the scheduler id list


    - #### getScheduler

      ```
      public WorkflowScheduler getScheduler(int schedulerId)
      ```

      Gets the scheduler .

      Parameters:
      :   `index` - the scheduler id

      Returns:
      :   the scheduler


- Overview
- Package
- Class
- Use
- Tree
- Deprecated
- Index
- Help

- Prev Class
- Next Class

- Frames
- No Frames

- All Classes

- Summary:
- Nested |
- Field |
- Constr |
- Method

- Detail:
- Field |
- Constr |
- Method
